# Supplementary material for: Immune and behavioral correlates of protection against symptomatic post-vaccination SARS-CoV-2 infection
Source: Front Immunol. 2024 Mar 19;15:1287504. doi: 10.3389/fimmu.2024.1287504 (PMC10985347; doi:10.3389/fimmu.2024.1287504)
Supplement: Supplementary file 1 [file DataSheet_1.pdf]

Supplementary figures (Immune and behavioral correlates of protection against symptomatic post-vaccination SARS-CoV-2 infection)

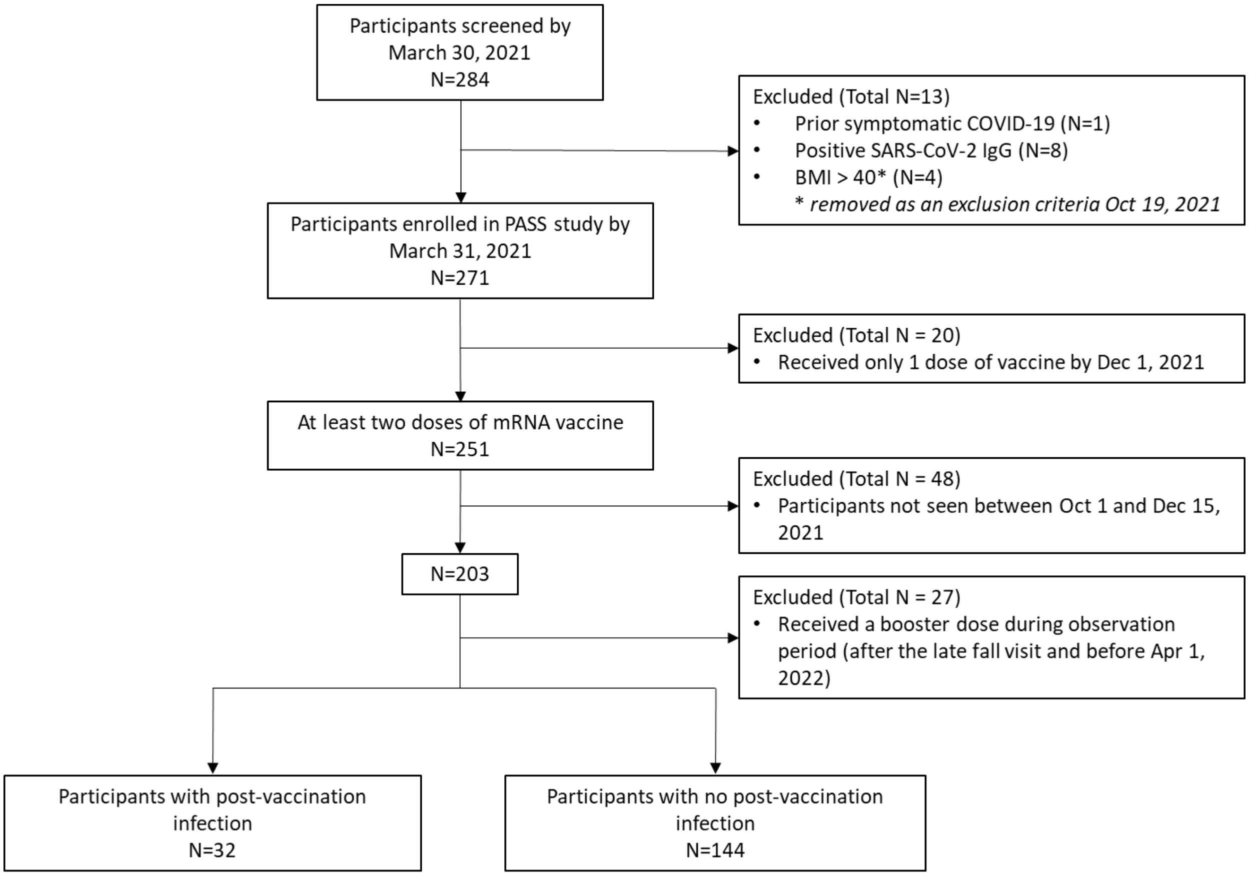

Supplementary Figure 1. Strobe chart of the study cohort

N represents the number of participants. BMI, Body Mass Index; PASS, Prospective Assessment of SARS-CoV-2 Seroconversion.

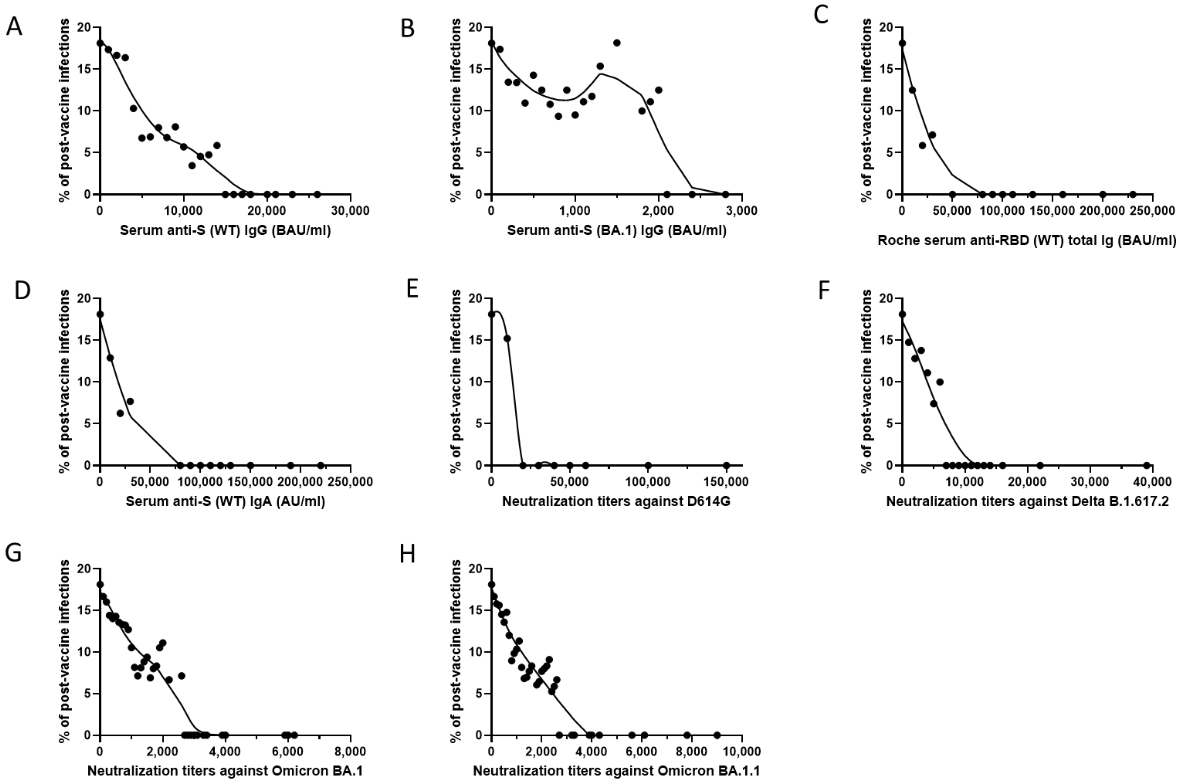

Supplementary Figure 2. ROC curves – Probability of post-vaccine SARS-CoV-2 infection as a function of immune markers

(A) Serum anti-S (WT) IgG measured by research assay. (B) Serum anti-S (BA.1) IgG measured by research assay. (C) Serum anti-RBD (WT) total Ig measured by Roche Elecsys® Anti-SARS-CoV-2 S assay. (D) Serum anti-S (WT) IgA measured by research assay. (E) Pseudovirus neutralization antibody titers against D614G. (F) Pseudovirus neutralization antibody titers against B.1.617.2. (G) Pseudovirus neutralization antibody titers against BA.1. (H) Pseudovirus neutralization antibody titers against BA.1.1.

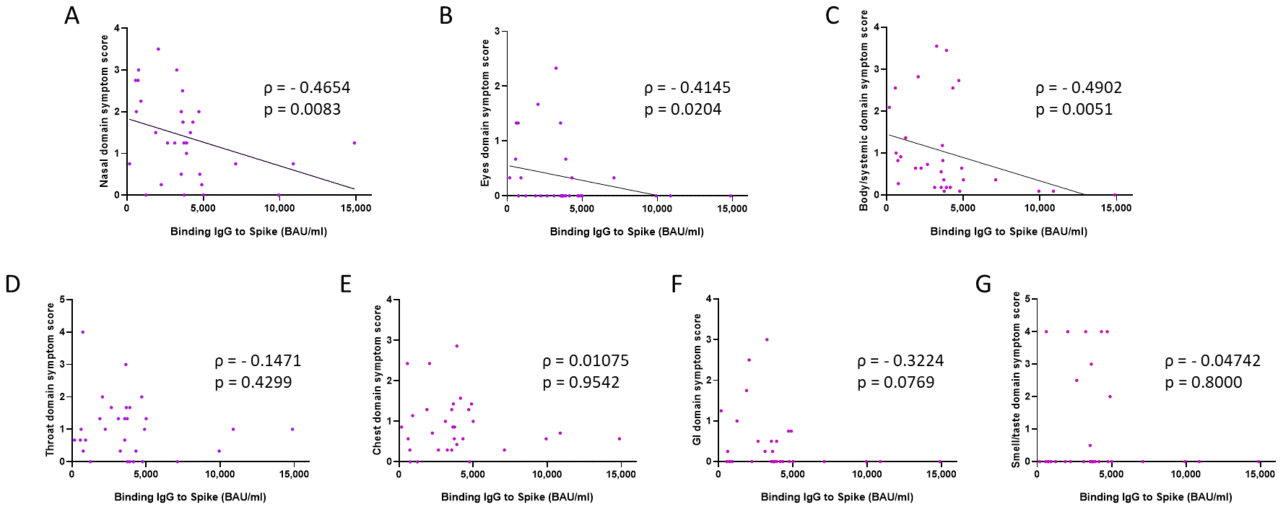

Supplementary Figure 3. Correlation plots of anti-S (WT) IgG serum levels and domain symptom scores in the PVI group

(A) Correlation between anti-S (WT) IgG serum levels (BAU/ml) and nasal domain symptom scores (Spearman ρ = -0.4654; p = 0.0083). (B) Correlation between anti-S (WT) IgG serum levels (BAU/ml) and eyes domain symptom scores (Spearman ρ = -0.4145; p = 0.0204). (C) Correlation between anti-S (WT) IgG serum levels (BAU/ml) and body/systemic domain symptom scores (Spearman ρ = -0.4902; p = 0.0051). (D) No correlation between anti-S (WT) IgG serum levels (BAU/ml) and throat domain symptom scores (Spearman ρ = -0.1471; p = 0.4299). (E) No correlation between anti-S (WT) IgG serum levels (BAU/ml) and chest domain symptom scores (Spearman ρ = 0.01075; p = 0.9542). (F) No correlation between anti-S (WT) IgG serum levels (BAU/ml) and gastrointestinal (GI) domain symptom scores (Spearman ρ = -0.3224; p = 0.0769). (G) No correlation between anti-S (WT) IgG serum levels (BAU/ml) and smell/taste domain symptom scores (Spearman ρ = -0.04742; p = 0.8). Dots indicate results from individual participants, n=32.

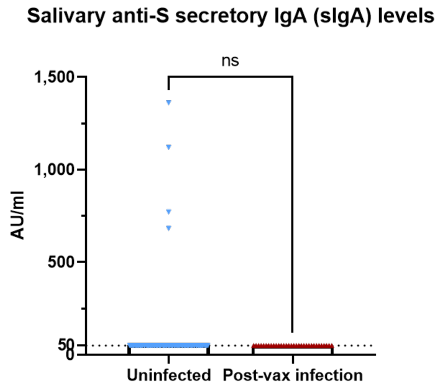

Supplementary Figure 4. Saliva anti-S (WT) secretory IgA levels

Saliva samples were obtained during the fall 2021 research clinic visit, at a median of 52.5 (IQR: 45.8 – 67.3) days after last immunization for the PVI group (n=32) and 46.5 (IQR: 32.8 – 58.3) days after last immunization for the uninfected group (n=143). A) Comparison of anti-S (WT) secretory IgA (sIgA) saliva levels between the uninfected group (n=143) and the PVI group (n=32). P values determined using the Mann-Whitney U test ($p = 0.5947$). Dots indicate results from individual participants and bars indicate geometric mean with 95% confidence intervals (CI). ns, not significant.

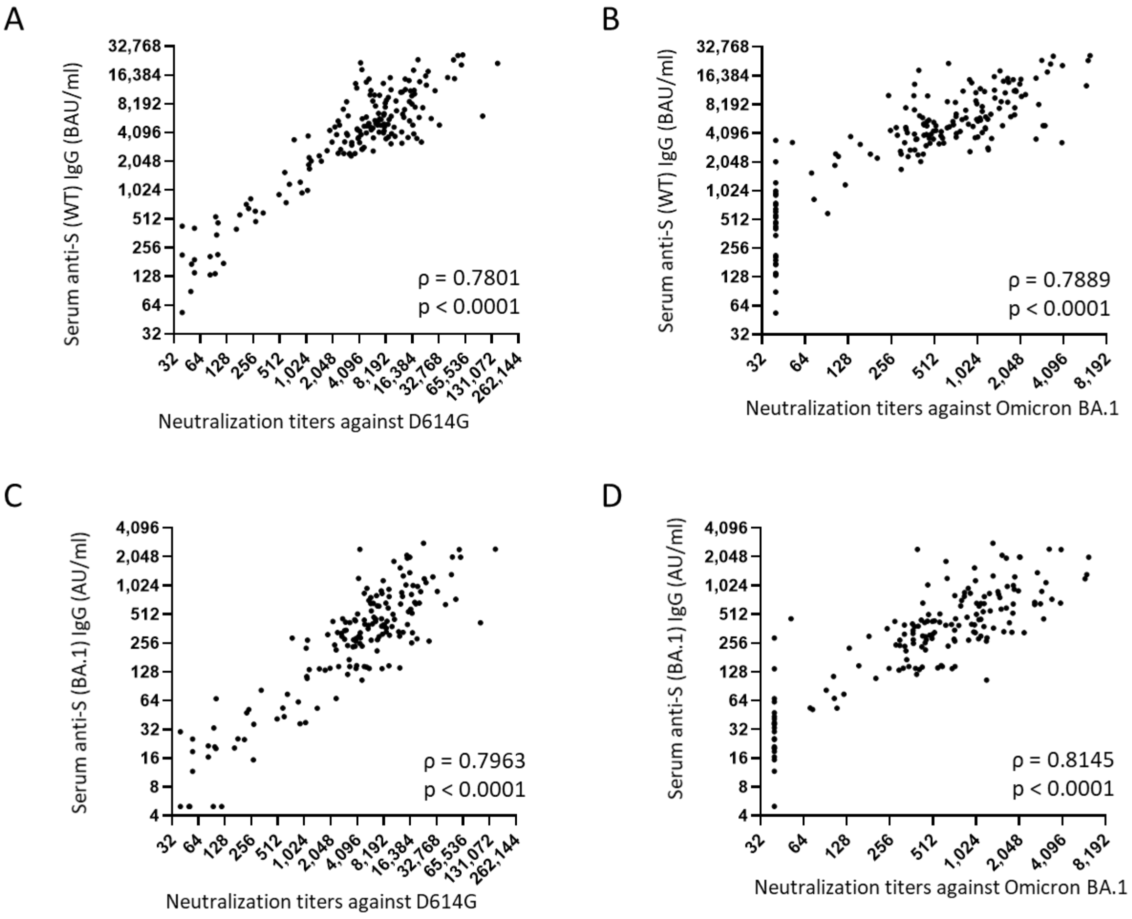

Supplementary Figure 5. Correlation plots of serum binding antibody levels against WT and BA.1 Spike and pseudovirus neutralization antibody titers against WT and BA.1

(A) Correlation between anti-S (WT) IgG serum levels (BAU/ml) and pseudovirus neutralization antibody titer (ID_{50}) against D614G (Spearman $\rho = 0.7801$; $p < 0.0001$). (B) Correlation between anti-S (WT) IgG serum levels (BAU/ml) and pseudovirus neutralization antibody titer (ID_{50}) against Omicron subvariant BA.1 (Spearman $\rho = 0.7889$; $p < 0.0001$). (C) Correlation between anti-S (BA.1) IgG serum levels (AU/ml) and pseudovirus neutralization antibody titer (ID_{50}) against D614G (Spearman $\rho = 0.7963$; $p < 0.0001$). (D) Correlation between anti-S (BA.1) IgG serum levels (AU/ml) and pseudovirus neutralization antibody titer (ID_{50}) against Omicron subvariant BA.1 (Spearman $\rho = 0.8145$; $p < 0.0001$). Dots indicate results from individual participants.

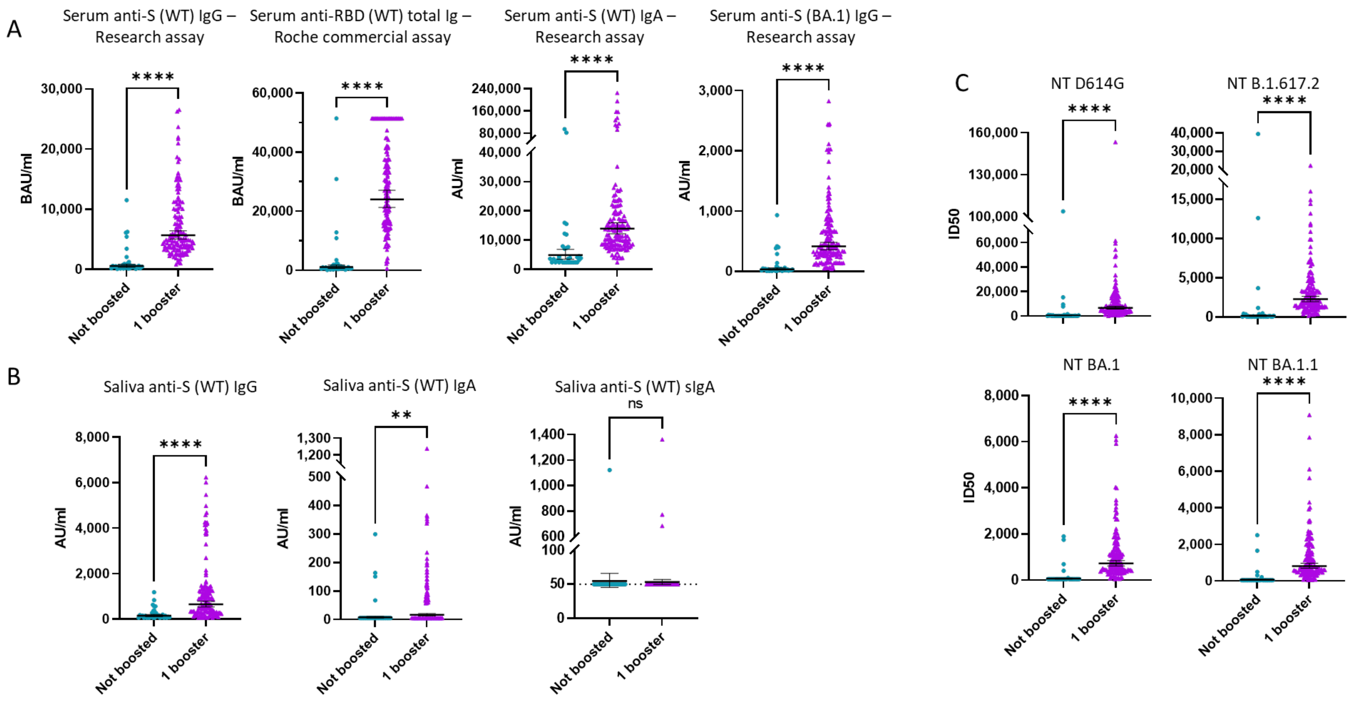

Supplementary Figure 6. Comparison of binding antibody serum levels, binding antibody saliva levels, and pseudovirus neutralization antibody titers between unboosted and boosted participants

(A) From left to right: comparison between unboosted and boosted participants of anti-S (WT) IgG serum levels (BAU/ml) measured with the research assay ($p < 0.0001$), anti-S (WT) total Ig serum levels (BAU/ml) measured with the Roche Elecsys® Anti-SARS-CoV-2 S assay ($p < 0.0001$), anti-S (WT) IgA serum levels (AU/ml) measured with the research assay ($p < 0.0001$), and anti-S (BA.1) IgG serum levels (AU/ml) measured with the research assay ($p < 0.0001$). (B) From left to right: comparison between unboosted and boosted participants of anti-S (WT) IgG saliva levels (AU/ml; $p < 0.0001$), anti-S (WT) IgA saliva levels (AU/ml; $p = 0.0079$), and anti-S (WT) sIgA saliva levels (AU/ml; $p = 0.7937$). (C) From left to right, top to bottom: comparison between unboosted and boosted participants of pseudovirus neutralization antibody titers (ID_{50}) against D614G ($p < 0.0001$), Delta variant B.1.617.2 ($p < 0.0001$), Omicron subvariant BA.1 ($p < 0.0001$), and Omicron subvariant BA.1.1 ($p < 0.0001$). P values determined using the Mann-Whitney U test. Dots indicate results from individual participants and bars indicate geometric mean with 95% CI. sIgA, secretory IgA. ** $p < 0.01$; **** $p < 0.0001$; ns, not significant.

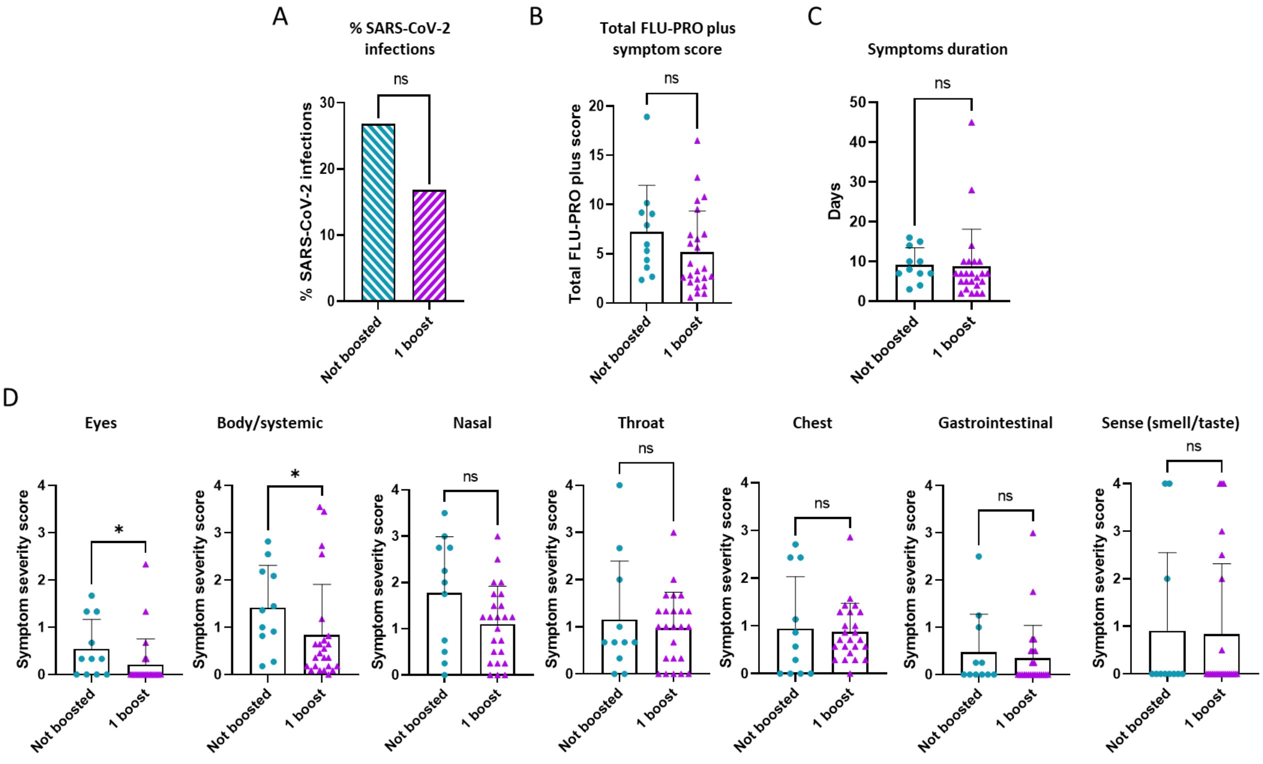

Supplementary Figure 7. Impact of boosting status on SARS-CoV-2 infection's likelihood, overall symptoms severity and duration, as well as domain symptom severity

(A) Percentage of infections in the unboosted versus boosted participants ($p = 0.1776$). (B) Total FLU-PRO plus symptom scores in the unboosted versus boosted participants ($p = 0.1628$). (C) Overall symptoms duration in the unboosted versus boosted participants ($p = 0.1496$). (D) Symptom severity scores for each symptom domain in the unboosted versus boosted participants (eyes, $p = 0.016$; body/systemic, $p = 0.0237$; nasal, $p = 0.1072$; throat, $p = 0.8517$; chest, $p = 0.5088$; gastrointestinal, $p = 0.6377$; sense, $p > 0.9999$). P values determined using the Mann-Whitney U test. Dots indicate results from individual participants and bars indicate mean with standard deviation. * $p < 0.05$; ns, not significant.

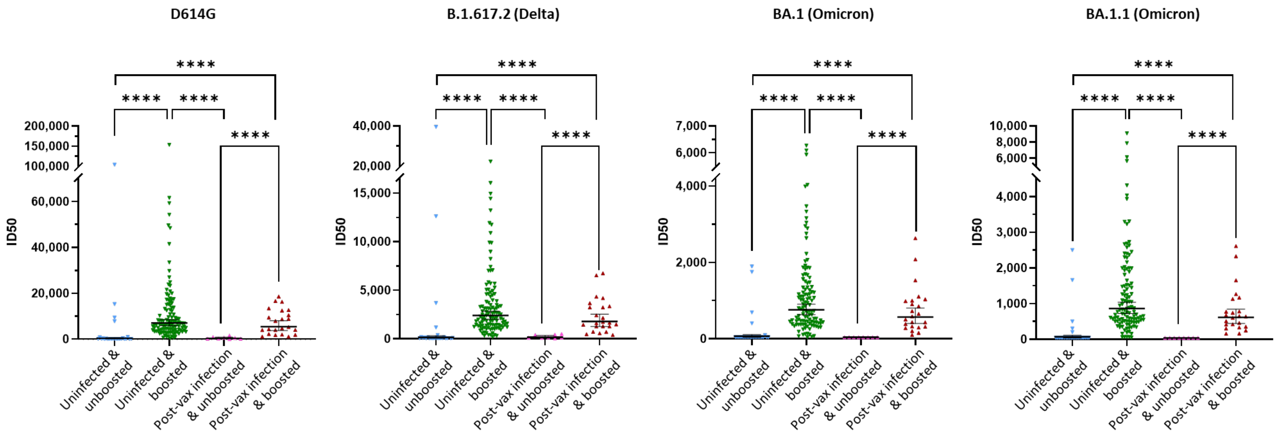

	D614G				B.1.617.2 (Delta)				BA.1 (Omicron)				BA.1.1 (Omicron)			
GMT	252	7037	348	5432	144	2396	168	1788	69	757	40	567	70	865	40	611

Supplementary Figure 8. Impact of boosting status on pseudovirus neutralization antibody titers in the uninfected versus post-vaccine infection group

Pseudovirus neutralization ID50 titers against D614G, Delta variant B.1.617.2, and Omicron subvariants BA.1 and BA.1.1 for uninfected & unboosted participants (N=25), uninfected & boosted participants (N=115), PVI & unboosted participants (N=8), and PVI & boosted participants (N=23). P values determined using the Mann-Whitney U test, with a Bonferroni correction for 6 comparisons, alpha = 0.0083. Dots indicate results from individual participants and bars indicate geometric mean titers (GMT) with 95% CI, and GMTs are indicated. **** p < 0.0001.

Work		Home	
**1. Risk Exposure**	**2. Precautionary Measures**	**3. Risk Exposure**	**4. Precautionary Measures**
1. Days working in the hospital (0-4)	1. Consistent PPE use during patient interactions (0-4)	1. Member of household tested COVID+ (0-4)	1. Mask wearing outside of the home (grocery stores) (0-4)
2. Direct contact with COVID+ patients (0-4)	2. PPE use when not directly interacting with patients (0-4)	2. Member of household had symptoms consistent with COVID and hasn't been tested (0-2)	2. Mask wearing while outside doing solitary activities (0-4)
3. Conducted high-risk activities on COVID+ patients (0-4)	3. Consistency of applying hand sanitizer/washing hands before and after interacting with patients (0-4)	3. Number of times in an out of house activity (grocery, restaurant, gym, bus…) (0-4 averaged for all 6 responses to this question)	3. Consistently practicing social distancing outside home (0-4)
		4. Size and number of social gatherings (0-4 averaged for all 5 responses to this question)	4. Disinfecting mail/packages delivered to home (0-4)
			5. Type of mask typically used (1-4 averaged for all 4 responses to this question)
**Work Risk Score= (Total score/12)x100**	**Work Precautionary Score= (Total score/12)x100**	**Home Risk Score= (Total score/14)x100**	**Home Precautionary Score= (Total score/20)x100**

Supplementary Table 1. Calculation method for the Work Risk Score, Work Precautionary Score, Home Risk Score, and Home Precautionary Score

These four scores are generated each month when a PASS participant fills out the “Risk Exposure, PPE Use, and Social Distancing” questionnaire. PPE, Personal Protective Equipment.

Sample	Location	Collection date	# Raw Reads	# Joined Reads Mapped	Consensus genome length (nt)	Average Coverage (x)	Pangolin Lineage	Nextstrain Clade
176	Bethesda, MD	01/2022	629,614	281,007	29,747	3,765	BA.1.20 (Omicron)	21K (Omicron)/high
177	Bethesda, MD	02/2022	737,940	331,425	29,747	4,432	BA.1.1 (Omicron)	21K (Omicron)/high
181	Bethesda, MD	12/2021	1,055,928	449,639	29,747	6,036	BA.1.1 (Omicron)	21K (Omicron)/high
182	Bethesda, MD	01/2022	619,074	260,449	29,747	3,468	BA.1.1 (Omicron)	21K (Omicron)/high
183	Bethesda, MD	01/2022	1,111,572	472,861	29,747	6,327	BA.1.20 (Omicron)	21K (Omicron)/high
184	Bethesda, MD	12/2021	634,940	269,282	29,747	3,600	BA.1.1 (Omicron)	21K (Omicron)/high
185	Bethesda, MD	12/2021	589,156	250,817	29,764	3,370	AY.25 (Delta)	21J (Delta)/high
186	Bethesda, MD	12/2021	1,001,634	412,655	29,708	5,563	BA.1.18 (Omicron)	21K (Omicron)/high
187	Bethesda, MD	12/2021	643,086	271,882	29,747	3,622	BA.1.15 (Omicron)	21K (Omicron)/high
188	Bethesda, MD	12/2021	714,018	299,757	29,747	3,960	BA.1.1 (Omicron)	21K (Omicron)/high
189	Bethesda, MD	12/2021	717,104	307,496	29,708	4,105	BA.1.18 (Omicron)	21K (Omicron)/high
190	Bethesda, MD	12/2021	465,830	197,371	29,747	2,587	BA.1.1 (Omicron)	21K (Omicron)/high
191	Bethesda, MD	01/2022	769,504	326,309	29,747	4,357	BA.1.19 (Omicron)	21K (Omicron)/high

Supplementary Table 2. Sequencing statistics for 13 SARS-CoV-2 post-vaccine infections

All samples produced coding complete genomes. Viral Amplicon Illumina Workflow 2.3 was used to collate and analyze SARS-CoV-2 genomes from the resulting sequencing reads. Consensus genomes were generated when possible. Lineage determination of consensus genomes was conducted using Pangolin (Phylogenetic Assignment of Named Global Outbreak LINeages; v4.1.2). Nextstrain clades were determined by Nextclade CLI 2.4.0, Nextalign CLI 1.10.1. Nextstrain overall sequence QC scores of ‘bad’, ‘mediocre’, and ‘good’ were translated into ‘low’, ‘medium’, and ‘high’ for confidence of clade assignment.

	Unadjusted	
	OR (95% CI)	P-value
Age	0.973 (0.826-1.147)	0.746
Female	0.406 (0.185-0.893)	0.025
Serum anti-S (WT) IgG (BAU/ml)	0.703 (0.527-0.937)	0.016
Serum anti-S (BA.1) IgG (AU/ml)	0.811 (0.616-1.067)	0.134
Serum anti-S (WT) IgA (AU/ml)	0.489 (0.180-1.329)	0.161
Roche Serum anti-RBD (WT) total Ig (BAU/ml)	0.479 (0.177-1.299)	0.148
Neutralizing titer D614G (ID50)	0.480 (0.197-1.166)	0.105
Neutralizing titer B.1.617.2 (ID50)	0.498 (0.236-1.050)	0.067
Neutralizing titer BA.1 (ID50)	0.694 (0.477-1.010)	0.056
Neutralizing titer BA.1.1 (ID50)	0.573 (0.341-0.961)	0.035
Saliva anti-S (WT) IgG (AU/ml)	1.051 (0.884-1.249)	0.576
Saliva anti-S (WT) IgA (AU/ml)	0.867 (0.521-1.443)	0.583
Work risk score (%)	1.074 (0.921-1.253)	0.362
Work precautionary score (%)	0.998 (0.844-1.180)	0.979
Home risk score (%)	2.144 (1.645-2.794)	0.000
Home risk score 1-2 (%)	1.760 (1.472-2.103)	0.000
Home risk score 3-4 (%)	1.044 (0.869-1.253)	0.647
Home precautionary score (%)	1.009 (0.842-1.209)	0.923
	**Adjusted for age, sex and home risk score**	
	**OR (95% CI)**	**P-value**
Serum anti-S (WT) IgG (BAU/ml)	0.673 (0.477-0.950)	0.025
Serum anti-S (BA.1) IgG (AU/ml)	0.785 (0.572-1.079)	0.136
Serum anti-S (WT) IgA (AU/ml)	0.428 (0.109-1.680)	0.224
Roche Serum anti-RBD (WT) total Ig (BAU/ml)	0.426 (0.109-1.662)	0.219
Neutralizing titer D614G (ID50)	0.350 (0.109-1.124)	0.078
Neutralizing titer B.1.617.2 (ID50)	0.409 (0.151-1.111)	0.080
Neutralizing titer BA.1 (ID50)	0.705 (0.445-1.117)	0.137
Neutralizing titer BA.1.1 (ID50)	0.511 (0.256-1.018)	0.056
Saliva anti-S (WT) IgG (AU/ml)	1.014 (0.800-1.285)	0.908
Saliva anti-S (WT) IgA (AU/ml)	0.851 (0.414-1.749)	0.661

Supplementary Table 3. Numeric values of point estimates and 95% CI for the odds ratios unadjusted and adjusted for non-immunological factors
